# Distributed RSS-Based Localization in Wireless Sensor Networks Based on Second-Order Cone Programming

**DOI:** 10.3390/s141018410

**Published:** 2014-10-01

**Authors:** Slavisa Tomic, Marko Beko, Rui Dinis

**Affiliations:** 1 Institute for Systems and Robotics (ISR), Instituto Superior Técnico (IST), Av. Rovisco Pais 1, Lisbon 1049-001, Portugal; 2 Universidade Lusófona de Humanidades e Tecnologias, Campo Grande 376, Lisboa 1749-024, Portugal; E-Mail: beko.marko@ulusofona.pt; 3 UNINOVA, Campus da FCT/UNL, Monte de Caparica, Caparica 2829-516, Portugal; 4 Instituto de Telecomunicações, Av. Rovisco Pais 1, Torre Norte, piso 10, Lisboa 1049-001, Portugal; E-Mail: rdinis@fct.unl.pt; 5 DEE-FCT, Universidade Nova de Lisboa, Monte de Caparica 2829-516, Portugal

**Keywords:** wireless localization, wireless sensor network (WSN), received signal strength (RSS), second-order cone programming (SOCP) problem, cooperative localization, distributed localization

## Abstract

In this paper, we propose a new approach based on convex optimization to address the received signal strength (RSS)-based cooperative localization problem in wireless sensor networks (WSNs). By using iterative procedures and measurements between two adjacent nodes in the network exclusively, each target node determines its own position locally. The localization problem is formulated using the maximum likelihood (ML) criterion, since ML-based solutions have the property of being asymptotically efficient. To overcome the non-convexity of the ML optimization problem, we employ the appropriate convex relaxation technique leading to second-order cone programming (SOCP). Additionally, a simple heuristic approach for improving the convergence of the proposed scheme for the case when the transmit power is known is introduced. Furthermore, we provide details about the computational complexity and energy consumption of the considered approaches. Our simulation results show that the proposed approach outperforms the existing ones in terms of the estimation accuracy for more than 1.5 m. Moreover, the new approach requires a lower number of iterations to converge, and consequently, it is likely to preserve energy in all presented scenarios, in comparison to the state-of-the-art approaches.

## 1. Introduction

Wireless sensor networks (WSNs) find applications in the most varied areas, such as monitoring (industrial, healthcare, environmental), energy-efficient routing, exploration (deep water, underground, outer space), surveillance, and many more [1]. A WSN comprises a number of sensor nodes (in the further text, called nodes), which are deployed over a region of interest in order to acquire the desired measurements. Besides sensing, nodes have the capability of communicating and processing the acquired data. Recent advances in radio frequency (RF) and micro-electro-mechanical systems (MEMS) permit the use of large-scale networks with hundreds or thousands of nodes [1]. WSNs are subject to changes in topology (e.g., adding nodes, node or link failures), which aggravates the development of even the simplest algorithms.

In many practical applications, data acquired inside a WSN are irrelevant if the referred location is not known. Accurate positioning of objects and people in both indoor and outdoor environments enables new applications in emergency and commercial services that can improve safety and efficiency in everyday life [2]. Hence, accurate information about nodes' positions is a valuable resource, which offers additional knowledge to the user.

Nodes are commonly deployed over a monitored area with limited to non-existing control of their position in space (e.g., thrown out of an aeroplane). Installing a global positioning system (GPS) device in each node would severely raise the network costs and restrict its applicability [1]. In order to reduce the implementation costs, only a small fraction of nodes are equipped with GPS, called anchor (reference) nodes. The remaining ones, called target nodes, determine their positions by using a kind of localization scheme that exploits the known positions of the anchor nodes [1,3].

Localization schemes typically rely on distance measurements between nodes. These measurements are extracted from the time-of-arrival (TOA), time-difference-of-arrival (TDOA), angle-of-arrival (AOA), round-trip time (RTT), received signal strength (RSS) information or a combination of them, depending on the available hardware. Even though less accurate than TOA or TDOA, RSS measurements are very attractive to system designers, due to the low device cost [4], which is why they caught much attention in the research society recently [5–12].

The required processing inherent to localization schemes can be executed in a centralized or distributed fashion. The former approach assumes the existence of a central processor (node or a base station) which gathers all measurements via wireless transmissions and solves the localization problem [5–8]. However, in large-scale networks, it is very likely that there would exist a bottleneck at and near the central processor, causing high energy drain [4]. Additionally, the computational complexity of a centralized approach is highly affected by the network size. Many applications do not have a central processor or none with enough computational capacity to handle the necessary calculations. In some practical applications, privacy may prohibit sharing objective functions between nodes [13] and a distributed approach becomes an interesting alternative [1,3]. This approach is characterized by low computational complexity and high-scalability, which makes it a preferable solution for large-scale and highly-dense networks [3]. However, distributed algorithms are executed iteratively, which raises the energy consumption and makes them sensitive to error propagation. In general, when the necessary number of iterations (until convergence) is lower than the average number of hops to the central processor, the distributed approach is likely to preserve energy [1].

In [10], a distributed RSS-based localization algorithm for WSNs was presented. This algorithm was characterized by a spatial constraint that limits the solution space to a region around the current position estimate. Using discretization of the solution space, the authors in [10] found the position update of each node by minimizing a local objective function over the candidate set using direct substitution. Although the computational characteristics of such algorithms are excellent, their performance highly depends on good initialization, since the objective function is non-convex and the algorithm may get trapped into a local minimum or a saddle point, causing a large estimation error. Another distributed approach using consensus and convex optimization for sensor node localization based on RSS measurements was introduced in [11]. This algorithm employed the semidefinite relaxation technique to transform the non-convex maximum likelihood (ML) estimator into a convex one. Béjar and Zazo showed in [11] that applying a distributed algorithm based on an augmented Lagrangian approach using primal-dual decomposition to the derived convex estimator converges to the solution of the centralized approach. However, the approach in [11] deals only with a non-cooperative localization problem, where a single target node emits beacon frames to all anchor nodes in the network. In [12], a distributed cooperative localization algorithm that dynamically estimates the path loss exponent (PLE) by using RSS measurements was introduced. In order to reduce the energy consumption and possibly improve the estimation accuracy, the authors in [12] presented a node selection mechanism to limit the number of cooperating neighbors. Bel *et al.* used a Gauss–Seidel approach where the PLE is fixed first, and a gradient descent search is applied to estimate the target positions, which are then used to update the estimate of the PLE. This approach is efficient in the sense of computational complexity, but its performance highly depends on good initialization and the step size of the search.

All mentioned approaches deal only with the localization problem, where the target's transmit power, *P_T_*, is known. In this paper, we design a novel distributed cooperative algorithm based on second-order cone programming (SOCP) relaxation for node localization in WSNs using RSS measurements. In huge contrast to the existing work, we investigate both cases of known and unknown *P_T_*. Hence, the main contribution of our work is two-fold. In the case where *P_T_* is known, the main contribution of our work is the reduction of the estimation error by more than 1.5 m in comparison to the state-of-the-art approaches. In the case where *P_T_* is not known, to the best of the authors' knowledge, there is no existing solution proposed to overcome the mentioned problem; hence, the main contribution of our work is a novel distributed SOCP-based algorithm for target node localization in the presence of unknown *P_T_* in a cooperative network. For both cases, we start by coloring the network to establish a working hierarchy inside the network. To color the network, we employ the second-order coloring scheme [14], which implies that no node has the same color as any of its neighbors nor its neighbors' neighbors. This way, we guarantee that our algorithm is completely distributed and efficient (collision-free and fast as nodes with the same color can work in parallel). Next, we break down the non-convex and computationally complex ML estimation problem into smaller sub-problems, *i.e.*, we pose the local ML estimation problem for each target node. We derive a non-convex estimator, which tightly approximates the local ML estimator for small noise. The SOCP relaxation technique is applied to the derived non-convex estimator in order to form a convex one, which can be efficiently solved by interior-point algorithms [15]. Each target node obtains the solution locally, using an iterative procedure. In the case where *P_T_* is not known, we propose a simple algorithm based on SOCP relaxation, which can be described in four parts. First, target nodes obtain the estimates of their positions by iteratively solving the proposed SOCP problem for unknown *P_T_.* Next, the position estimates are fixed and used to attain an estimation of *P_T_* at each target node. Target nodes then obtain the average estimated value of *P_T_* by means of average consensus. Finally, target nodes update their position estimates by solving the proposed SOCP problem for known *P_T_*, by exploiting the consensus estimate of *P_T_.* In addition, a possible improvement of the convergence properties of the proposed algorithm for known *P_T_* is investigated, by allowing a first-degree memory in the nodes. We propose a simple heuristic approach, which combines the previous and the new target position estimates in order to derive new estimates closer to the true target locations. At last, we provide details about the computational complexity and energy consumption of the considered algorithms.

The remainder of the paper is structured as follows. In Section 2 the RSS measurement model for cooperative localization is introduced, and the ML optimization problem for locating multiple target nodes is formulated. Section 3 provides details about the development of the proposed SOCP estimators for both cases of known and unknown *P_T_.* The computational complexity and energy consumption analysis are summarized in Section 4. In Section 5, we provide the computational complexity, energy consumption and simulation results to compare the performance of the newly proposed estimators with the existing ones. Finally, in Section 6, we summarize the main conclusions.

## 2. Problem Formulation

We consider a large-scale WSN with *M* target and *N* anchor nodes, randomly deployed over a region of interest. The considered network can be seen as a connected graph, 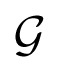
( 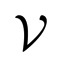, 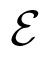, with | 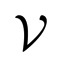
| = *M* + *N* vertices and | 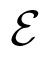
| edges, where | • | represents the cardinality of a set. The set of target nodes and the set of anchor nodes are labeled as 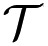 = {*t* : *t* = 1,…, *M*} and 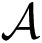 = {*a : a* = *M* + 1,…, *M* + *N*}, respectively. Without loss of generality, this paper focuses on the 2D scenario (the extension to 3D scenario is straightforward), where the true locations of the nodes are denoted as ***x****_i_* (***x****_i_* ∈ ℝ^2^), ∀*_i_ ε* 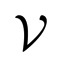 ( 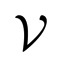 = 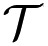 ∪︀ 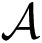)*.* Due to battery consumption (the lifetime of the network), it is assumed that all nodes have limited communication range, *R* (the generalization to other cases is straightforward). Hence, two nodes, *i* and *j*, can exchange information if and only if they are within the communication range of each other. The set of connections (edges) is defined as 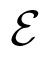 = {(*i*, *j*) : ‖*x_i_* — *x_j_*‖ ≤ *R*, *i*∈ 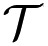,*j*∈ 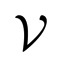,*i*≠*j*}*.* Accordingly, the set of neighbors of target node *i* is defined as 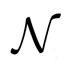
*_i_* = {*j* : (*i*,*j*) *ε*∈}*.*

For ease of expression, let us define *X* = [*x*_1_,*x*_2_, …,*x_m_*] as the matrix of all target node positions that need to be determined (***X*** ∈ ℝ^2x^*^M^*). We assume that the anchor positions are known *a priori*, while each target node *i* is given an initial estimation of its position, ${\hat{x}}_{i}^{\left(0\right)}$ , ∀*_i_* ∈ 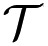; hence, *X^* ^(0)^ contains all initial target node position estimations. Furthermore, sets 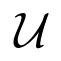 = {(*i*,*j*) : ‖*x_i_* — *x_j_*‖ ≤ *R*, ∀*i* ∈ 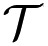, ∀*_j_* ∈ 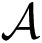} and 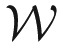 = {(*i*, *k*) : ‖***x****_i_* — **x***_k_*‖ ≤ *R*, ∀*i*,*k* ∈ 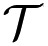, *i* ≠ *k*} denote the existence of the target/anchor and the target/target connections, respectively.

Using the relationship $L_{ij}\left(\text{dB}\right)=10\log_{10}\frac{P_{T}}{P_{ij}}$ , where *L_ij_* is the path loss between two nodes *i* and *j*, *P_T_* is the transmission power of a node, and *P_ij_* is the received power at a distance ‖***x****_i_* — *x_j_*‖ from the transmitting node, it is easy to see that the localization problem can be formulated by the path loss instead of the RSS. Under the log-normal shadowing and log-distance path loss model, the path loss between two nodes *i* and *j* can be modeled according to the following radio propagation path loss model (in dB) [16,17]:

(1) $$\begin{array}{c} L_{ij}^{u}=L_{0}+10\gamma \log_{10}\frac{\left\|x_{i}-x_{j}\right\|}{d_{0}}+v_{ij},\left(i,j\right)\in u\\ L_{ik}^{W}=L_{0}+10\gamma \log_{10}\frac{\left\|x_{i}-x_{k}\right\|}{d_{0}}+v_{ik},\left(i,k\right)\in W \end{array}$$

where *L*_0_ is the path loss measured at a short reference distance *d*_0_ (‖***x****_i_* — *x_j_* ‖ ≥ *d*_0_) from the transmitting node, *γ* is the PLE and *υ_ij_* is the log-normal shadowing term between the *i*-th and *j*-th node, modeled as a zero-mean Gaussian random variable with variance $\sigma_{ij}^{2}$ , *i.e.*, $\upsilon_{ij}\sim N\left(0,\sigma_{ij}^{2}\right)$ . We assume that the target/target path loss measurements are symmetric, *i.e.*, $L_{ik}^{W}=L_{ki}^{W}$ for *i* ≠ *k.*

Given the observation vector ***L*** = [*L_ij_*] (***L*** ∈ ℝ^|^ *^∈^*^|^), the conditional probability density function (pdf) is:

(2) $$\begin{array}{c} p\left(L|X\right)=\prod_{\left(i,j\right):\left(i,j\right)\in u}\frac{1}{\sqrt{2\pi \sigma_{ij}^{2}}}\exp-\frac{{\left(L_{ij}^{u}-L_{0}-10\gamma \log_{10}\frac{\left\|x_{i}-x_{j}\right\|}{d_{0}}\right)}^{2}}{2\sigma_{ij}^{2}}\\ +\prod_{\left(i,k\right):\left(i,k\right)\in W}\frac{1}{\sqrt{2\pi \sigma_{ik}^{2}}}\exp-\frac{{\left(L_{ik}^{W}-L_{0}-10\gamma \log_{10}\frac{\left\|x_{i}-x_{k}\right\|}{d_{0}}\right)}^{2}}{2\sigma_{ik}^{2}} \end{array}$$

The most common estimator used in practice is the ML estimator, since it has the property of being asymptotically efficient (for large enough data records) [18]. The ML estimator forms its estimate as the matrix ***X***, which maximizes the conditional pdf in Equation (2); hence, the ML estimator is obtained as:

(3) $$\begin{array}{c} \hat{X}=\underset{X}{\arg\min}=\sum_{\left(i,j\right):\left(i,j\right)\in u}\frac{1}{\sigma_{ij}^{2}}{\left[\left(L_{ij}^{u}-L_{0}\right)-10\gamma \log_{10}\frac{\left\|x_{i}-x_{j}\right\|}{d_{0}}\right]}^{2}\\ +{\sum_{\left(i,k\right):\left(i,k\right)\in W}\frac{1}{\sigma_{ik}^{2}}\left[\left(L_{ik}^{W}-L_{0}\right)-10\gamma \log_{10}\frac{\left\|x_{i}-x_{k}\right\|}{d_{0}}\right]}^{2} \end{array}$$

Even though the ML estimator is approximately the minimum variance unbiased (MVU) estimator [18], the least squares (LS) problem in Equation (3) is non-convex and has no closed-form solution. In the remainder of this work, we will show that the LS problem in Equation (3) can be relaxed as a SOCP, which can be solved efficiently by interior-point algorithms [15].

### 2.1. Assumptions

Before we proceed, let us prompt some assumptions made about the considered WSN:

(1) $\sigma_{ij}^{2}=\sigma^{2}$ , ∀ (*i*,*j*) ϵ 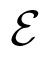;
(2) All nodes are equipped with omnidirectional antennas and have identical *P_T_*;
(3) The network is connected and it does not vary in time;
(4) A coloring scheme of the network is available.

**Figure 1.**
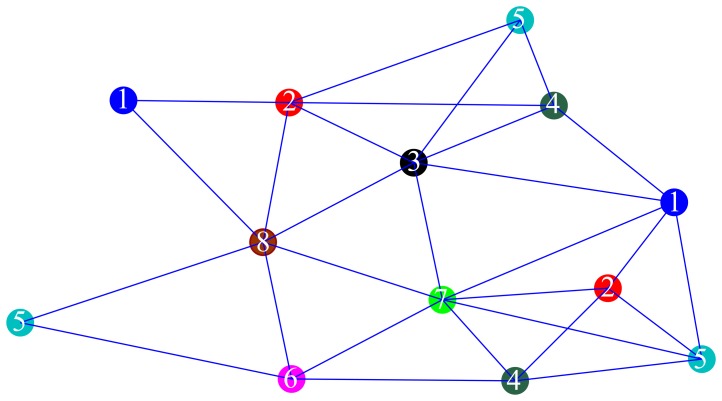
A possible second-order coloring scheme for a network with | 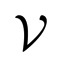
| = 13 nodes.

Assumptions (1) and (2) are made for the sake of simplicity (without loss of generality). Assumption (2) implies that *L*_0_ and *R* are equal for all nodes. In Assumption (3), a network is connected if and only if there exists a path between each nodes *i*,*j* ϵ 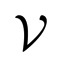. Finally, in Assumption (4), a coloring scheme is an assignment of colors (numbers) to the nodes in order to establish a working hierarchy in the network. In this work, we employ a second-order coloring scheme, meaning that no node has the same color (number) as any of its neighbors nor its neighbors' neighbors [14,19], as illustrated in Figure 1.

As one can see from Figure 1, the blue nodes will be the first ones to estimate their positions, while all other nodes are in the idle state. After the blue nodes finish working, they will broadcast the updated position to their neighbors. The red nodes will be the next ones to work, using the updated information from the blue nodes, and so on, following the colors (numbers) in Figure 1. Each node in the network needs to know only its own color and after which color (neighbor) is its turn to operate, rather than all the colors in the network. Note that, since we employed the second-order coloring scheme, nodes with the same color may work in parallel without the risk of message collision at the receiving end. According to Figure 1, the execution time of the algorithm is decreased from the time needed to realize | 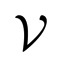
| = 13 operations to | 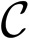
| = 8 operations by working in parallel, where 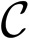 represents the set of colors of the nodes. Note that the network coloring problem may be considered as an optimization problem where the goal is to minimize the number of different colors. Although interesting in its own right, we did not investigate this problem here, since it does not follow the main idea of this work. In wireless scenarios, coloring schemes are used in media access control (MAC) protocols in order to achieve collision-free algorithms [19].

## 3. Distributed Approach Using SOCP Relaxation

Note that the objective function in (3) depends only on the positions and pairwise measurements between the adjacent nodes. Assuming that the initial position estimations of the target nodes are known, the problem in Equation (3) can be partitioned, *i.e.*, the minimization can be executed independently by each target node, using only the information gathered from its neighbors. Hence, instead of solving Equation (3), which can be computationally exhausting (in large-scale WSNs), we break down Equation (3) into subproblems, which are solved locally (by each target node) using the iterative approach. Therefore, target node *i* updates its position estimate by minimizing the following local ML problem:

(4) $${\hat{x}}_{i}^{\left(k+1\right)}=\underset{x_{i}}{\arg\min}\sum_{j\in N_{i}}\frac{1}{\sigma^{2}}{\left[L_{ij}-L_{0}-10\gamma \log_{10}\frac{\left\|x_{i}-{\hat{x}}_{j}\right\|}{d_{0}}\right]}^{2},\forall i\in T$$

where *x^_j_* denotes the last position update of the *j*-th neighbor (if the *j*-th neighbor is a target node) or the true neighbor's position (if the *j*-th neighbor is an anchor node) received by the *i*-th target node. From Equation (4), we distinguish two possible cases of the localization problem: *P_T_* of the transmitting node is known; and a more practical scenario, where *P_T_* is not known at the receiving end. In the following text, we deal with these two cases, and we propose a distributed solution based on the SOCP relaxation for both of them.

### 3.1. Transmit Power Is Known

From the propagation model (1), the estimated distance between the *i*-th target node and its *j*-th neighbor, *d^_ij_*, is given by:

(5) $${\hat{d}}_{ij}\approx d_{0}10^{\frac{L_{ij}-L_{0}}{10\gamma }}$$

We can rewrite Equation (5) as:

(6) $$\lambda_{ij}{\hat{d}}_{ij}\approx \alpha d_{0}$$

where $\lambda_{ij}=10^{-\frac{L_{ij}}{10\gamma }}$ and $\alpha =10^{-\frac{L_{0}}{10\gamma }}$. Assuming that the initial target position estimates are available, from Equation (6), the updated position of the *i*-th target node, ${\hat{x}}_{i}^{\left(k+1\right)}$, is found by minimizing the following LS problem. Another possible explanation with more theoretical justification leading to Equation (7) is as follows. We can rewrite Equation (1) as $\frac{L_{i}-L_{0}}{10\gamma }=\log_{10}\frac{\left\|x_{i}-x_{j}\right\|}{d_{0}}+\frac{\nu_{ij}}{10\gamma }$ which corresponds to $\lambda_{ij}\left\|x_{i}-x_{j}\right\|=\alpha d_{0}10^{\frac{v_{ij}}{10\gamma }}$ *.* For sufficiently small noise, the first-order Taylor series expansion to the right-hand side of the previous expression is given by $\lambda_{ij}\left\|x_{i}-x_{j}\right\|=\alpha d_{0}\left(1+\frac{\ln10}{10\gamma }\upsilon_{ij}\right)$ , *i.e.*, *λ_ij_* ‖***x****_i_* — ***x****_j_* ‖ = *αd*_0_ + *ε_ij_*, where ${ɛ}_{ij}=\alpha d_{0}\frac{\ln10}{10\gamma }\upsilon_{ij}$ is the zero-mean Gaussian random variable with the variance $\alpha^{2}d_{0}^{2}\frac{{\left(\ln10\right)}^{2}}{100\gamma^{2}}$ *.* Clearly, the corresponding LS estimator is given by Equation (7). A similar processhas been done in [8]. Even though our estimator is derived under the assumption that the noise is small, it works excellent in the case where this assumption does not hold, as we will see in Section 5:

(7) $${\hat{x}}_{i}^{\left(k+1\right)}=\underset{x_{i}}{\arg\min}\sum_{j\in N_{i}}{\left(\lambda_{ij}\left\|x_{i}-{\hat{x}}_{j}\right\|-\alpha d_{0}\right)}^{2},\forall i\in T$$

The LS problem in Equation (7) is non-convex and has no closed-form solution. However, in the following text, we will show that Equation (7) can be written as a convex optimization problem by using SOCP relaxation. First, define auxiliary variables *d_ij_* = ‖***x****_i_* — *x^_j_*‖ and *z* = [*z_ij_*], where *z_ij_* = *λ_ij_d_ij_* — *αd*_0_, ∀ (*i*, *j*) ∈ 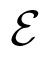 We get:

$$\underset{x_{i}d_{ij},z}{\min\text{imize}}\sum_{j\in N_{i}}z_{ij}^{2}$$

subject to:

(8) $$z_{ij}=\lambda_{ij}d_{ij}-\alpha d_{0},d_{ij}=\left\|x_{i}-{\hat{x}}_{j}\right\|$$

Introduce an epigraph variable, *t.* By using the second-order cone constraint (SOCC) relaxation, we obtain the following convex optimization problem:

$$\begin{array}{cc} \underset{x_{i},d_{ij},z,t}{\min\text{imize}} & t \end{array}$$

subject to:

(9) $$\begin{array}{ccc} \left[2z;t-1\right]\leq t+1, & z_{ij}=\lambda_{ij}d_{ij}-\alpha d_{0}, & \left\|x_{i}-{\hat{x}}_{j}\right\|\leq d_{ij} \end{array}$$

Problem (9) is an SOCP problem, which can be efficiently solved by the CVX package [20] for specifying and solving convex programs. Note that by applying the SOCC relaxation, the original set of feasible points is enlarged, which means that the optimal solution of Equation (9), $x_{i}^{*}$ might not be the optimal solution of Equation (7). However, if the SOCC of the form ‖*p*‖ ≤ *q* is satisfied as an equality, we have that the SOCC relaxation is not a relaxation at all and that $x_{i}^{*}$ of Equation (9) is also the optimal solution of Equation (7).

**Figure 2.**
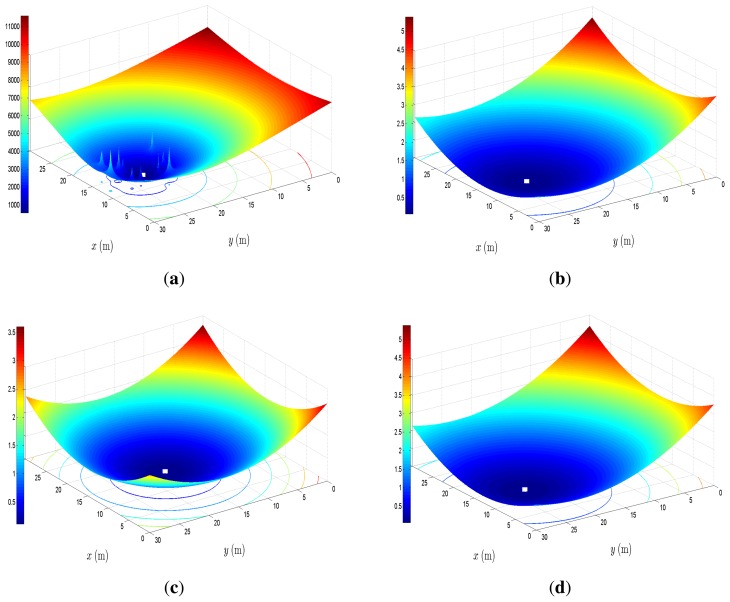
Illustration of the cost functions (4) and (7) versus *x* and *y* coordinates (target location); the minimum of the cost function is indicated by a white square. (**a**) Objective function in Equation (4) using the true target positions; (**b**) objective function in Equation (7) using the true target positions; (**c**) objective function in Equation (7) after one iteration; (**d**) objective function in Equation (7) after ten iterations.

In summary, the derivation of the above SOCP approach can be described in two parts. In the first part, the local non-convex ML estimator in Equation (4) is approximated by a different non-convex estimator in Equation (7). The use of the objective function in Equation (7) is motivated by the fact that we get a much smoother surface in comparison to Equation (4), at a cost of introducing some bias with respect to the ML solution (see Figure 2). If the bias effect is small, we might reach the ML solution by employing a local search around the solution of Equation (7). In the second part of our approach, we convert Equation (7) into a convex problem by applying SOCP relaxation.

Figure 2 illustrates a realization of the objective functions in Equations (4) and (7), for the case where the true node positions are used (Figure 2a,b) and a realization of Equation (7) when the estimated positions of the target nodes, obtained by solving Equation (9), are used: after the first and the tenth iteration (Figure 2c,d), respectively. We randomly placed *M* = 50 target and *N* = 25 anchor nodes inside a square region of the size 30 × 30 m^2^. The i-th target node is located at [12.8; 23.0] and has five anchor and five target nodes as its neighbors. All target nodes were given an initial guess of their positions in the center of the mentioned area. The rest of the parameters are set to: *L*_0_ = 40 dB, σ = 0 dB, γ = 3; and the objective functions are plotted *versus x* and *y* coordinates (the step in the mesh grid is 0.1 m). Figure 2a shows that the objective function in Equation (4), given the true positions of the target nodes, has a global minimum at [12.8; 23], and some local minima and saddle points. Figure 2b shows that the objective function in Equation (7), given the true positions of the target nodes, has a global minimum at [12.8; 23.1] and is much smoother than Equation (4). We can see that the objective functions Equations (4) and (7) have similar behavior: both monotonically increase and decrease in the same regions. In Figure 2c, one can see that the global minimum has shifted to [14.4; 17.3]. Furthermore, it can be seen that the shape of this objective function does not coincide with the objective function in Figure 2b. This is due to the fact that in the first iteration, target nodes broadcast their initial position guess to their neighbors, which results in high estimation error. However, as the number of iterations is increased, the estimation accuracy becomes better, as shown in Figure 2d, where the global minimum is located at [12.8; 23]. From Figure 2, it is clear that the objective function in (7) is an excellent approximation of the objective function in Equation (4).

Algorithm 1 summarizes the proposed distributed SOCP approach for known *P_T_.* Algorithm 1 is distributed in the sense that no central node coordinates the network, all communication occur exclusively between two incident nodes and the data associated with each node is processed locally. Lines 5–7 are executed simultaneously by all nodes *i* ∈ 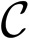
*_c_*, which may decrease the execution time of the algorithm. The only information exchange occurs in Line 7, when the nodes broadcast their position updates ${\hat{x}}_{i}^{\left(k+1\right)}$ to their neighbors. Since ${\hat{x}}_{i}^{\left(k+1\right)}$ , we can conclude that the proposed algorithm requires at most a broadcast of **2***K*_max_*M* real values.


**Algorithm 1** “SOCP1”: the proposed distributed second-order cone programming (SOCP) algorithm for known *P_T_*.
**Require: *X^***
^(0)^ , *K*_max_, 

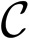
, ***x****_a_***,** ∀*_a_* ∈ 

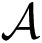
1:**Initialize:**
*k* ← 12:**repeat**3:** for**
*c* = 1,…**,

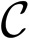
 do**4:**  for all**
*i* ∈ 

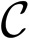
*_c_* (in parallel) **do**5:   Collect *x^_j_* from *j* ∈ 

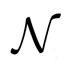
*_i_*6:   
${\hat{x}}_{i}^{\left(k\right)}$ ← solve Equation (9)7:   Broadcast
${\hat{x}}_{i}^{\left(k\right)}$ to *j* ∈ 

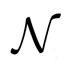
*_i_*8:**  end for**9:** end for**10:*k* ← *k* + 111:**until**
*k* ≤ *K*_max_


### 3.2. Transmit Power Is Not Known

In order to minimize the expenses and achieve a low-cost localization system, testing and calibration of the devices are usually not the priority in practice [1]. This means that the node transmit power, *P_T_*, is not calibrated, *i.e.*, not known. Not knowing *P_T_* corresponds to not knowing *L*_0_ in the path loss model; hence, in this subsection, *L*_0_ is considered to be an unknown parameter that also needs to be estimated. Adaptation of the SOCP approach for known *L*_0_ is straightforward for the case where *L*_0_ is not known:

$$\begin{array}{cc} \underset{x_{i},d_{ij},z,t,\alpha }{\text{minimize}} & t \end{array}$$

subject to:

(10) $$\begin{array}{ccc} \left[2z;t-1\right]\leq t+1, & z_{ij}=\lambda_{ij}d_{ij}-\alpha d_{0}, & \left\|x_{i}-{\hat{x}}_{j}\right\|\leq d_{ij} \end{array}$$

Algorithm 2 summarizes the proposed distributed SOCP approach for unknown *P_T_.* This algorithm can be easily explained in four parts. The first part includes Lines 1–12, where we estimate the targets' positions by solving the SOCP problem described in Equation (10)*K*_1max_ number of times. In order to minimize the oscillations in the position estimates, we employ a weight, *w*, in Line 7 of our algorithm. Although the difference in the performance is marginal, according to our simulations, the best choice of the weight is $w=\frac{1}{\sqrt{k}}$ . Then, in Lines 13–17, we use the estimates obtained in the *K*_1max_-th iteration to find the estimate of *L*__0__, *L^*__0__*_i_*, at each target node. Since we assumed that *L*__0__ is identical for all nodes, an average consensus is executed next in order to reach the average estimated value of *L*__0__, *L*_0_*;* lines 18–20. To realize the average consensus, we employ the local-degree weights method [21], since it is particularly suitable for distributed implementation and guarantees convergence (if the graph is not bipartite). The final part of our algorithm involves Lines 21–31, which are executed K_2max_ number of times. Here, we take advantage of *L^*_0_ and obtain the targets' position estimates by solving Equation (9) as if *L*_0_ is known, *i.e.*, we calculate $\hat{\alpha }=10^{-\frac{{\hat{L}}_{0}}{10\gamma }}$ . Concerning the information exchange of the proposed algorithm, it is easy to see that it requires at most a broadcast of (2 (*K*_1max_ + *K*_2max_) + *D*_max_) *M* real values, given that *D*_max_ is the maximum number of communications between nodes in order to reach the consensus.

## 4. Energy Consumption Analysis

To evaluate the overall performance of an algorithm, besides the estimation accuracy, it is also important to asses its energy consumption. The trade-off between the estimation accuracy and the energy consumption determines the applicability potential of an algorithm; thus, it is its key feature.

To prolong the lifetime of a network, *i.e.*, to maintain the lifetime of nodes' battery, it is very important to mitigate the energy depletion in a WSN. Two major energy-consuming phases of an algorithm are: data processing and data communication. In the following text, we provide an analysis of these two phases. However, in order to provide a more complete overview of the algorithms' performance, we present the results of the analysis together with the simulation results in Section 5.

### 4.1. Data Processing

As we mentioned earlier, data collected inside the network is processed locally by each target node in a distributed approach. In order to evaluate the efficiency of an approach, in terms of data processing, we have to determine its computational complexity. The formula for computing the worst case computational complexity of a SOCP problem [22], given below, is used to calculate the complexity of the proposed approach:

(11) $$O\left(\sqrt{L}\left(m^{2}\sum_{i=1}^{L}n_{i}+\sum_{i=1}^{L}n_{i}^{2}+m^{3}\right)\right)$$

where *L* is the number of the second-order cone constraints, *m* is the number of the equality constraints and *n_i_* is the dimension of the *i*-th second-order cone.

Let us assume that we can always equalize the energy consumption of two algorithms in the data processing phase through voltage adjustment [23]. Energy depletion of two algorithms is then compared through the execution time of the algorithms, meaning that a more energy-efficient algorithm is the more time-efficient one.

### 4.2. Data Communication

After processing the data in order to update its position estimate, a node immediately broadcasts this estimate to its neighbors. This phase of the algorithm is the most expensive, regarding the energy-consumption, since the energy required to transmit one bit could be used to execute thousands of instructions, depending on the hardware and the range [1,4].


**Algorithm 2** “SOCP2”: the proposed distributed SOCP algorithm for unknown *P_T_*.
**Require:**
***X^***
^(0)^ , *K*_1max_, *K*_2max_, 

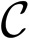
, *x_a_*, ∀*a* ∈ 

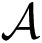
1:**Initialize:**
*k* ← 12:**repeat**3:** for**
*c* = 1, …,**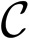 do**4:**  for all**
*i* ∈ 

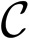
*_c_* (in parallel) **do**5:   Collect *x^_j_* from *j* ∈ 

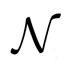
*_i_*6:   
${\hat{x}}_{i\text{socp}}^{\left(k\right)}$ ← solve (10)7:   
${\hat{x}}_{i}^{\left(k\right)}\leftarrow \left(1-w\right){\hat{x}}_{i}^{\left(k-1\right)}+w{\hat{x}}_{i\text{socp}}^{\left(k\right)}$8:   Broadcast
${\hat{x}}_{i}^{\left(k\right)}$ to *j* ∈ 

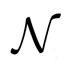
*_i_*9:**  end for**10:** end for**11: *k* ← *k* + 112:**until**
*k* ≤ *K*_1max_13:**for**
*c* = 1, …,**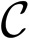 do**14:**  for all**
*i* ∈ 

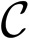
*_c_* (in parallel) **do**15:  
${\hat{L}}_{0i}\leftarrow \frac{\sum_{j\in N_{i}}L_{ij}-10\gamma \log_{10}\frac{\left\|{\hat{x}}_{i}^{\left(k\right)}-{\hat{x}}_{j}^{\left(k\right)}\right\|}{d_{0}}}{\left|N_{i}\right|}$16:** end for**17:**end for**18:begin **consensus**19:
${\hat{L}}_{0}\leftarrow \frac{1}{M}\sum_{i=1}^{M}{\hat{L}}_{0i}$20:end **consensus**21:*k* ← *K*_1max_ + 122:**repeat**23:** for**
*c* = 1, …,**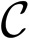 do**24:**  for all**
*i* ∈ 

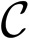
*_c_* (in parallel) **do**25:   Collect *x^_j_* from *j* ∈ 

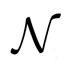
*_i_*26:   
${\hat{x}}_{i}^{\left(k\right)}$ ← solve (9) by using *L^*_0_27:   Broadcast
$${\hat{x}}_{i}^{\left(k\right)}$$ to *j* ∈ 

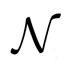
*_i_*28:**  end for**29:** end for**30: *k* ← *k* + 131:**until**
*k* ≤ *K*_1max_ + K_2max_


The formula for computing the total amount of energy consumed by the network in the communication phase, given below, is employed:

(12) $$E_{\text{com}}=\left(E_{T_{x}}M+E_{R_{x}}\sum_{i=1}^{M}\left|N_{i}\right|\right)K_{\text{req}}$$

where *E_Tx_* and *E_Rx_* denote the energy consumed by a node for broadcasting and receiving data, respectively, and *K*_req_ is the number of iterations required for an algorithm to converge.

## 5. Simulation Results

In this section, computer simulations are performed in order to compare the performance of the proposed approaches with the state-of-the-art. The proposed algorithms were solved by using the MATLAB package CVX [20], where the solver is SeDuMi [24].

A random deployment of *M* target and *N* anchor nodes inside a square region of length *B* in each Monte Carlo (*M_c_*) run is considered. The random deployment of the nodes is of particular interest, since a common practical requirement for a WSN is that it is flexible in topology; hence, the localization algorithms need to be robust to various scenarios. In order to make the comparison of the considered approaches as fair as possible, we first obtained *M_c_* = 500 target and anchor nodes positions, as well as noise realizations between nodes *(i*,*j)* ∈ 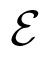 in each *M_c_* run. Furthermore, we made sure that the network graph is connected in each *M_c_* run. We then solved the localization problem with the considered approaches for those scenarios. In all simulations presented here, the path loss exponent is set to *γ* = 3, the reference distance *d*_0_ = 1 m, the power loss *L*_0_ = 40 dB and the communication range of a node is *R* = *B/*5 m. We assumed that the initial guess of the target positions, *X^* ^(0)^, is in the intersection of the diagonals of the square area, since the biggest possible error for this case is half of the diagonal of the area. In the case of known *P_t_*, the maximum number of iterations is set to *K_m_*_ax_ = 200, while for unknown *P_t_*, *K*_1max_ = 10 and *K*_2max_ = 25, unless stated otherwise. The performance metric is the normalized root mean square error (NRMSE), defined as:

$$\text{NRMSE}=\sqrt{\frac{1}{MM_{c}}\sum_{i=1}^{M_{c}}{\sum_{j=1}^{M}\left\|x_{ij}-{\hat{x}}_{ij}\right\|}^{2}}$$

where *x^_ij_* denotes the estimate of the true location of the *j*-th target, *x_ij_*, in the *i*-th Monte Carlo run.

In Table 1, we provide an overview of the considered algorithms in this section, together with their worst case computational complexities. To the best of authors' knowledge, the state-of-the-art algorithms for the case where *P_t_* is known are described in [10,12], whereas for the case when *P_t_* is not known, there is no existing work.

From Table 1, it is clear that the computational complexity of the distributed algorithms mainly depends on the size of the neighborhood fragments, rather than the total number of nodes in the WSN. Although it is theoretically possible to have that max{| 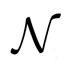
*_i_*|} = *M* + *N* — 1, for *i* = 1,…,*M*, in practice, the size of the neighborhood fragments are much smaller, due to limited *R.* For example, in our simulations, the majority of nodes had very few neighbors, and the average size of fragments was well below 10. Increasing the number of nodes in the network does not necessarily impact the neighborhood fragments (or not significantly), which is why the distributed algorithms are preferable in large-scale and highly-dense WSNs in contrast to the centralized ones. Table 1 also reveals that the proposed algorithms are computationally the most demanding, in comparison to the existing ones. However, this fact is justified by their superior performance in the estimation accuracy, as we will see in the following text. Additionally, it is important to stress that the communication process is much more energy-consuming than the computation one [1,4].

In [10], the authors proposed a discretization of the search region over a 5 × 5 resolution grid, since it represents the best trade-off between accuracy and computational complexity. In all scenarios considered here, for the approach in [10], we have discretized the search region with *F* = 100 randomly generated points in order to achieve higher estimation accuracy for this approach. The approach in [12] considers the localization problem where the PLE is not known. However, it is straightforward to adapt it for the case of known PLE, by using the true value of the PLE instead of the estimated one. In all considered scenarios, for the approach in [12], the step size of the gradient descent method is set to *γ_x_* = 10^−3^, while the required number of iterations are set to *t*_iter1_ = *K*_max_ and *t*_iter2_ = 5. Although in [12], the authors consider node selection mechanisms in order to determine the optimum number of cooperating nodes and obtain a good trade-off in terms of position accuracy *versus* energy consumption, we do not employ it here. The reason is that, for the chosen scenarios, the number of neighbors is below the optimum value for a majority of the target nodes; therefore, we allow target nodes to cooperate with all neighboring nodes, independent of the fragment size.

### 5.1. Known *P*_T_

**Figure 3.**
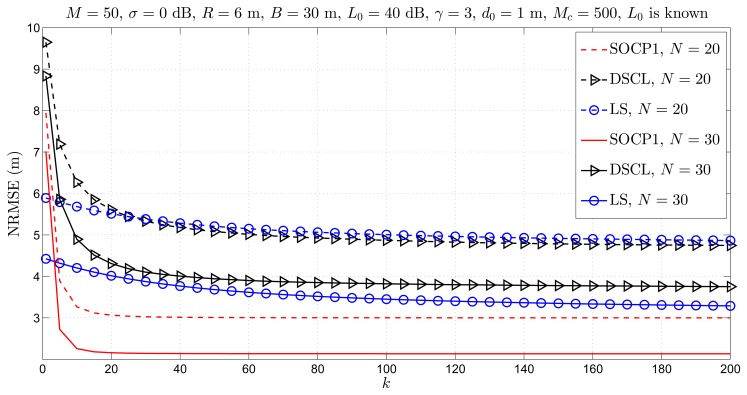
Simulation results for cooperative localization when *L*_0_ is known: normalized root mean square error (NRMSE) *versus k* comparison for different *N*, when *M* = 50, σ = 0 dB, *R* = 6 m, *B* = 30 m, *L*_0_ = 40 dB, γ = 3, *d*_0_ = 1 m, *M_c_* = 500.

Figure 3 illustrates the NRMSE *versus k* performance of the considered approaches for different *N.* From Figure 3, we can see that the estimation accuracy of all approaches better outcomesas *k* and/or *N* increases, as anticipated. Moreover, it can be seen that the proposed approach outperforms the existing ones in both estimation accuracy and convergence sense. In terms of the estimation accuracy, the new approach reduces the estimation error on average for about 1.5–2 m, when compared to the existing ones. In terms of convergence, one can see that the proposed approach requires only *k* = 20 iterations to converge, while state-of-the-art approaches do not converge even after *k* = 200 iterations. This result is very important in the sense of energy conservation, since the proposed approach may be stopped after only *k* = 15 iterations, while the existing ones require much more iterations. Finally, it is worth mentioning that the “LS” approach outperforms the “DSCL” and the proposed one in the first iteration. This is due to the fact that the “LS” approach uses a weighted mean of the coordinates of the *n* nearest anchor nodes to determine the initial guess of the targets' positions. This method, as well as the other ones, such as multidimensional scaling (MDS) [25], can also be used to obtain the initial guess for the proposed approach. However, these methods increase the computational cost, and we do not use them for our approach.

**Figure 4.**
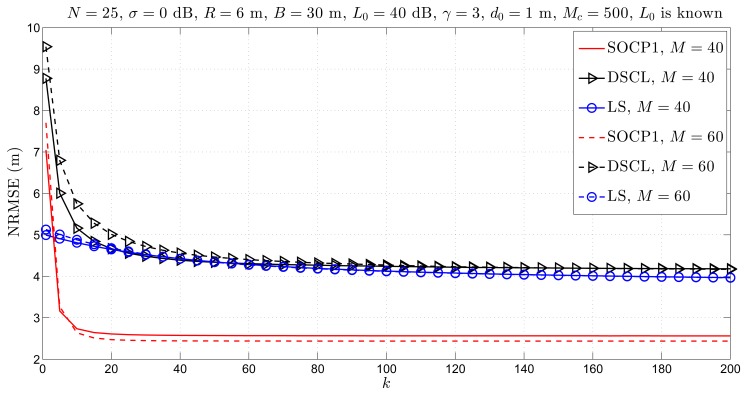
Simulation results for cooperative localization when *L*_0_ is known: NRMSE *versus k* comparison for different *M*, when *N* = 25, *σ* = 0 dB, *R* = 6 m, *B* = 30 m, *L*_0_ = 40 dB, γ = 3, *d*_0_ = 1 m, *M_c_* = 500.

Figure 4 illustrates the NRMSE *versus k* performance of the considered approaches for different *M.* From Figure 4, it can be seen that all approaches require slightly higher number of iterations when *M* is increased. However, the estimation accuracy of all approaches does not deteriorate when more target nodes are added in the network; the proposed approach performs even better when *M* increases. Additionally, Figure 4 exhibits superior performance of the proposed approach in both estimation accuracy and convergence realizations, in comparison to state-of-the-art. We can see that the new approach improves the estimation accuracy for more than 1.5 m, thereby requiring not more than *k* = 20 iterations to converge, while the existing methods require at least *k* = 120 iterations to converge.

**Figure 5.**
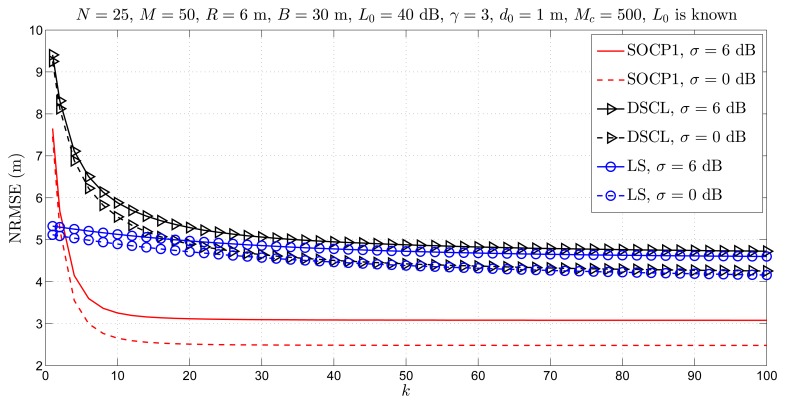
Simulation results for cooperative localization when *L*_0_ is known: NRMSE *versus k* comparison for different *σ*, when *N* = 25, *M* = 50, *R* = 6 m, *B* = 30 m, *L*_0_ = 40 dB, γ = 3, *d*_0_ = 1 m, *K*_max_ = 100, *M_c_* = 500.

In Figures 3 and 4, we investigated the case where the wireless channel is noise-free. In practice, however, the channel is prone to noise influence, which can severely impact the algorithm's performance. Hence, in Figure 5, we investigate the influence of noise on the performance of the considered algorithms.

Figure 5 illustrates the NRMSE *versus k* performance of the considered approaches for *σ* = 6 dB. As the performance benchmark, we employ the simulation results of the considered methods when the channel is noise-free, *i.e.*, *σ* = 0 dB in this figure. From Figure 5, one can see that as *σ* increases, the performance of all approaches deteriorates by approximately 0.5 m. Moreover, we can see that the new approach converges after only *k* = 20 iterations for both choices of *σ*, while the existing approaches do not converge after *k* = *K_m_*_ax_ number of iterations. Finally, Figure 5 shows that the proposed approach outperforms the state-of-the-art in terms of the estimation accuracy, achieving a gain of more than 1.5 m for both choices of *σ.*

**Figure 6.**
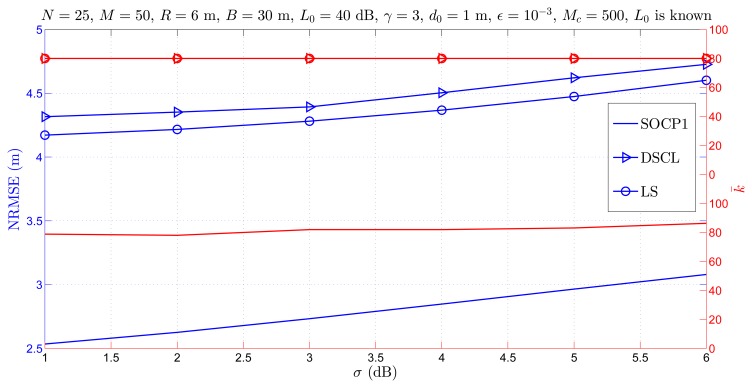
Simulation results for cooperative localization when *L*_0_ is known: NRMSE and *k̄ versus σ* comparison, when *N* = 25, *M* = 50 dB, *R* = 6 m, *B* = 30 m, *L*_0_ = 40 dB, γ = 3, *d*_0_ = 1 m, *K*_max_ = 100, *ε* = 10^−3^, *M_c_* = 500.

From Figures 3, 4 and 5, one can conclude that perhaps it is not necessary for an algorithm to perform all *K*_max_ number of iterations, since we might preserve energy if less iterations are performed. Thus, in Figure 6, we illustrate the NRMSE performance of the considered approaches for different *σ*, as well as the average number of required iteration, *k̄*, when a stopping criterion, $\left\|{\hat{x}}_{i}^{\left(k+1\right)}-{\hat{x}}_{i}^{\left(k\right)}\right\|\leq ɛ$ , is introduced. This stopping criterion implies that a target node will stop calculating its position estimate if two consecutive estimations of its position are sufficiently close to each other. Once this condition is met by all target nodes, the network will stop working in order to save energy.

In Figure 6, blue solid lines represent the NRMSE performance (left *y*-axis) and red solid lines represent *k̄* performance (right *y*-axis) of the considered approaches for different *σ*, when *K*_max_ = 100 and *ε* = 10^−3^. As can be seen from Figure 6, the new approach outperforms the state-of-the-art by more than 1.5 m for all ranges of *σ.* Furthermore, we can see that the proposed approach also outperforms the existing ones in terms of convergence. Our approach requires on average about *k̄* = 42 iterations to converge, while the existing approaches do not meet the stopping condition in *K*_max_ number of iterations.

Let us interpret the above result in terms of energy consumption. In Table 2, we represent the average running time per node per *M_c_* run of the considered algorithms for *M_c_* = 100 runs, which we will use to analyze the energy consumption of the algorithms.

Assuming that *E*_proc_ is the total energy required for processing the data, *E_p_* is the energy spent per working second in data processing phase, *t* is the average running time of an algorithm, *K*_req_ = *k*, $E_{c}=E_{T_{x}}M+E_{R_{x}}\sum_{i=1}^{M}\left|N_{i}\right|$ is the energy spent per iteration in data communication phase, we present the results for the energy depletion of the considered algorithms in Table 3.

From Table 3, one can see that the proposed approach is the most energy consuming in the data processing phase. This result is expected, since the proposed approach is more computationally complex, in comparison to the existing ones. Furthermore, we can see that our approach preserves almost 60% of energy in the communication phase, in comparison to state-of-the-art. Since the communication phase is much more energy expensive than the data processing one [1,4], *i.e.*, *E_c_* ≫ *E_p_*, we can conclude that the proposed approach is likely to preserve energy for all values of *σ.* Moreover, since our approach requires a significantly lower number of signal transmissions, the utilization efficiency of the radio spectrum can be enhanced. This result is important, because the radio spectrum is a precious resource for wireless communications.

#### 5.1.1. Heuristic Approach for Improving the Convergence of the Proposed Algorithm

Although the proposed algorithm converges in less iterations than the existing ones, we have observed in our simulations that it still has potential for further improvement. In the following text, we give a short overview of the proposed heuristic approach, which improves the convergence of the proposed algorithm for known *P_t_*, without a significant increase in the computational complexity.

Target node *i* first obtains a solution of the SOCP problem in Equation (9), ${\hat{x}}_{i\text{socp}}^{\left(k+1\right)}$ *.* Then, the target node takes an additional step from the point ${\hat{x}}_{i\text{socp}}^{\left(k+1\right)}$ in the direction $\vec{{\hat{x}}_{i}^{\left(k\right)}{\hat{x}}_{i\text{socp}}^{\left(k+1\right)}}$ *.* The size of the step taken is $w\delta_{i}^{\left(k+1\right)}$ , where *w* is the weight used to avoid oscillations in the position estimates and:

$$\delta_{i}^{\left(k+1\right)}=\frac{\sum_{j\in N_{i}}\left|r_{ij}-\left\|{\hat{x}}_{i}^{\left(k\right)}-{\hat{x}}_{j}\right\|\right|}{\left|N_{i}\right|}$$

given that $r_{ij}=d_{0}10^{\frac{L_{ij}-L_{0}}{10\gamma }}$ . The motivation for calculating the step size in this way is that by improving the position estimates in every iteration, we are also minimizing the difference between the measured and the Euclidean distances; therefore, we are decreasing the step size in each iteration. Hence, an intermediate estimate is obtained as:

$${\hat{x}}_{i\text{imp}}^{\left(k+1\right)}={\hat{x}}_{i\text{socp}}^{\left(k+1\right)}+\delta_{i}^{\left(k+1\right)}\left[\cos\left(\alpha_{i}\right);\sin\left(\alpha_{i}\right)\right]$$

where *α_i_* is the angle between points ${\hat{x}}_{i}^{\left(k\right)}$ and ${\hat{x}}_{i\text{socp}}^{\left(k+1\right)}$ *.* At last, target node *i* determines its updated position estimate as:

$${\hat{x}}_{i}^{\left(k+1\right)}=\left(1-w\right){\hat{x}}_{i\text{socp}}^{\left(k+1\right)}+w{\hat{x}}_{i\text{imp}}^{\left(k+1\right)}$$

and immediately broadcasts this estimate to its neighbors.

**Figure 7.**
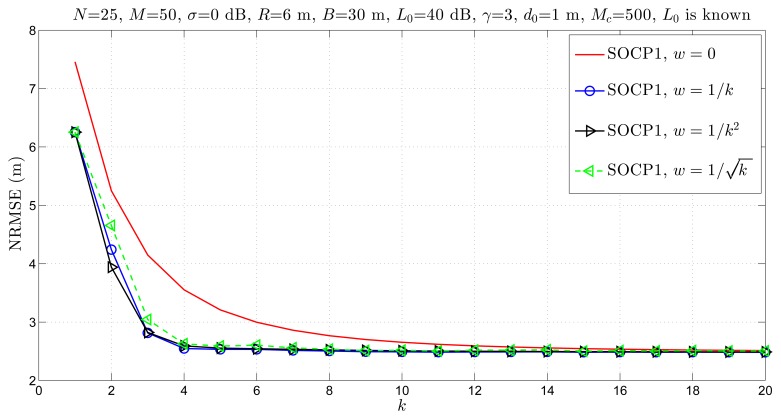
NRMSE *versus* number of iterations comparison of the proposed approach for different choices of *w*.

In Figure 7, we have investigated the influence of the chosen weight on the estimation accuracy of the proposed approach. Note that the choice *w* = 0 corresponds to the case where ${\hat{x}}_{i}^{\left(k+1\right)}={\hat{x}}_{i\text{socp}}^{\left(k+1\right)}$ , *i.e.*, no additional step is taken after the SOCP solution is attained. Figure 7 shows that a considerable gain in the estimation accuracy is achieved in the first few iterations by taking the additional step. Moreover, it can be seen that the new approach converges after only *k* = 5 iterations for the choice *w* ≠ 0, whereas *k* = 15 iterations are necessary for *w* = 0. Finally, even though the difference in the performance is marginal, based on our simulations, we have concluded that the best choice of the weight is $w=\frac{1}{k}$ in general.

**Figure 8.**
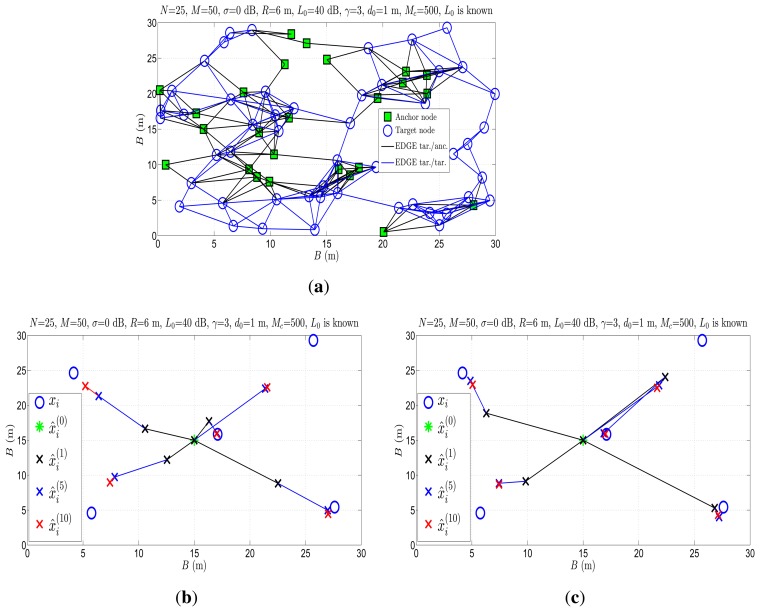
Estimation process of the proposed approach in the first 10 iterations. (**a**) Network configuration; (**b**) SOCP1 approach, *w* = 0; (**c**) SOCP1 approach, $w=\frac{1}{k}$.

Figure 8b,c illustrate an example of the estimation process in the first ten iterations for the proposed approach, when *w* = 0 and $w=\frac{1}{k}$, respectively. In Figure 8a, we show the network layout considered for this particular example.

Comparing Figure 8b,c, one can see that, in general, making an additional step of the length $\delta_{i}^{\left(k\right)}$ in the first iteration pushes the estimates much closer to the true target positions. As the number of iterations increases, the step size, *i.e.*, *w*, decreases, giving more importance to the solution obtained with the proposed SOCP approach. Additionally, from Figure 8, we can see that the target node, which has no anchor nodes as its neighbors (upper right corner), suffers the lowest, while the target node closest to ${\hat{x}}_{i}^{\left(0\right)}$ experiences the highest estimation accuracy in these few iterations. Although we cannot guarantee that our approach will converge under all conditions, our simulation results show that it is a good heuristic.

### 5.2. Unknown *P*_T_

**Figure 9.**
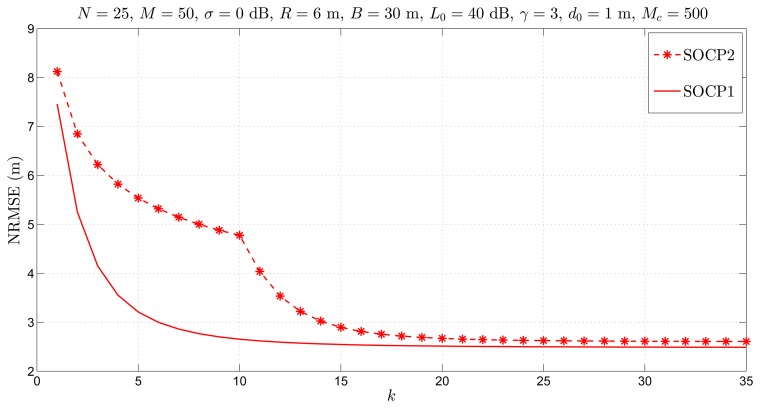
Simulation results for cooperative localization for known and unknown *L*__0__*:* NRMSE *versus k* comparison, when *N* = 25, *M* = 50, *σ* = 0 dB, *R* = 6 m, *B* = 30 m, *L*__0__ = 40 dB, *γ* = 3, *d*_0_ = 1 m, *M_c_* = 500.

Figure 9 illustrates the NRMSE *versus k* performance of the proposed approach for unknown *P_T_.* As the performance benchmark, we employ the proposed approach when *P_T_* is known in this figure, since we are not aware of any existing work that addresses the distributed localization problem for the case when *P_T_* is not known. From Figure 9, it can be seen that the proposed approach reduces the estimation error as *k* increases, as expected. Furthermore, the saddle point at *k* = 10 is observed. This is because, at this point, we obtain an estimate of *P_T_*, and we proceed with our algorithm as if *P_T_* is known, using its estimated value. From Figure 9, we can conclude that the proposed approach provides excellent estimation accuracy, since it attains the lower bound given by the results of the proposed approach for known *P_T_.*

## 6. Conclusions

In this work, we addressed the RSS-based target localization problem in a cooperative WSN. A novel distributed cooperative algorithm based on SOCP relaxation is presented, for both cases of known and unknown target transmit power, *P_T_*. For both scenarios, we start with the network coloring to ensure a completely distributed and collision-free algorithm. We derive the ML estimation problem to localize all target nodes simultaneously, and we break it down into local ML problems for each target node. The local ML estimator is tightly approximated by another non-convex estimator for small noise. The derived non-convex estimator allows the use of convex optimization tools in order to convert the estimation problem into a convex one. Hence, appropriate convex relaxations are applied to the derived non-convex estimator, and novel SOCP estimators are proposed to solve the target localization problem for known and unknown *P_T_*. As we considered a distributed implementation of the proposed algorithms, they are executed iteratively. In the case of unknown *P_T_* , we solve the localization problem *K*_1max_ number of times, after which we attain the ML estimate of *P_T_* by fixing the target estimates. We then take advantage of this estimate in order to further improve the estimation accuracy of the proposed approach; hence, we proceed in our algorithm as if *P_T_* is known. Additionally, we propose a simple heuristic approach to further improve the convergence of the proposed approach for known *P_T_*, which requires a first-degree memory in the target nodes. Moreover, we provided details about the computational complexity and energy consumption of the considered algorithms. The simulation results confirm the effectiveness of the proposed algorithms, showing a remarkable improvement in the estimation accuracy of more than 1.5 m in the case of known *P_T_*, in comparison with the state-of-the-art. The new approach requires less number of iterations to converge and is likely to preserve energy in all scenarios presented in this work. When *P_T_* is not known, the simulation results certify the excellent performance of our approach, attaining the lower bound defined by the new approach for known *P_T_*.

## Figures and Tables

**Table 1.** Summary of the considered algorithms.

**Algorithm**	**Description**	**Complexity**
DSCL	The spatially constrained algorithm in [10]	$K_{\max}\times M\times F\times O\left(\underset{i}{\max}\left\{\left|N_{i}\right|\right\}\right)$
LS	The least squares algorithm in [12]	$K_{\max}\times M\times t_{iter2}\times O\left(\underset{i}{\max}\left\{\left|N_{i}\right|\right\}\right)$
SOCP1	The proposed Algorithm 1 for known *P_T_*	$K_{\max}\times M\times O\left({\left(\underset{i}{\max}\left\{\left|N_{i}\right|\right\}\right)}^{3.5}\right)$
SOCP2	The proposed Algorithm 2 for unknown *P_T_*	$\left(K_{1\max}+K_{2\max}\right)\times M\times O\left({\left(\underset{i}{\max}\left\{\left|N_{i}\right|\right\}\right)}^{3.5}\right)+M\times D_{\max}$

**Table 2.** The average running time per node per *M_c_* run of the considered algorithms for known *P_T_*, when *N* = 25, *M* = 50, *σ* = 0 dB, *R* = 6 m, *M_c_* = 100. CPU: Intel(R)Core(TM)i7-363QM 2.40 GHz.

**Algorithm**	**Time (s)**
DSCL	0.0013
LS	0.0004
SOCP1	0.36

**Table 3.** The average energy depletion of the considered algorithms for known *P_t_*, when *N* = 25, *M* = 50, σ = 0 dB, *R* = 6 m, *M_c_* = 100.

**Algorithm**	*E*_proc_ (J)	*E*_com_ (J)	*E*tot (J) = *E*proc + *E*com
DSCL	*E_p_*×*t*×*M*× *K*_req_	*E_c_* × *K*_req_	63.4*E_p_* + 100*E_c_*
LS	*E_p_*×*t*×*M*× *K*_req_	*E_c_* × *K_req_*	21.7*E*_p_ + 100*E_c_*
SOCP1	*E_p_*×*t*×*M*× *K*_req_	*E_c_* × *K*_req_	746.48*E_p_* + 42*E_c_*
